# Mother-to-Infant Bonding in Women With a Bipolar Spectrum Disorder

**DOI:** 10.3389/fped.2021.646985

**Published:** 2021-03-19

**Authors:** Myrthe G. B. M. Boekhorst, Annemerle Beerthuizen, Manon Hillegers, Victor J. M. Pop, Veerle Bergink

**Affiliations:** ^1^Department of Psychiatry, Erasmus MC, University Medical Center, Rotterdam, Netherlands; ^2^Department of Medical and Clinical Psychology, Tilburg University, Tilburg, Netherlands; ^3^Department of Child and Adolescent Psychiatry and Psychology, Erasmus MC, University Medical Center, Rotterdam, Netherlands; ^4^Departments of Psychiatry and Obstetrics, Icahn School of Medicine at Mount Sinai, New York, NY, United States

**Keywords:** bonding, bipolar spectrum disorder, postpartum mania, perinatal, relapse

## Abstract

**Purpose:** Offspring of mothers with a bipolar disorder are at high-risk for impaired developmental outcomes and psychopathology (e. g., mood, anxiety, sleep disorders) later in life. This increased risk of psychopathology is not only because of genetic vulnerability, but environmental factors may play an important role as well. The often long and debilitating mood episodes of mothers with bipolar disorder might hamper their qualities as a caregiver and may impact the child. We examined early mother-to-infant bonding 1 year postpartum in mothers with bipolar spectrum disorder as compared to mothers of the general population. The association between mother-to-infant bonding and the type of bipolar spectrum diagnosis (bipolar I, bipolar II, bipolar Not Otherwise Specified) as well as relapse within 12 months postpartum was also assessed.

**Methods:** In total, 75 pregnant women with a bipolar spectrum disorder participated in the current study. The participants were included in a longitudinal cohort study of women with bipolar spectrum disorder and were prospectively followed from pregnancy until 1 year postpartum. Mother-to-infant bonding was assessed using the Pre- and Postnatal Bonding Scale. A longitudinal population-based cohort of 1,419 pregnant women served as the control group. Multiple linear regression analyses were used to assess the association between bipolar spectrum disorder and mother-to-infant bonding scores, controlling for several confounders.

**Results:** Women with bipolar spectrum disorder perceived the bonding with their child as less positive compared to the control group. The type of bipolar spectrum disorder was not associated with poorer bonding scores. Relapse during the 1st year after delivery also did not affect bonding scores in women with bipolar spectrum disorder.

**Conclusions:** Our findings could imply that women with bipolar spectrum disorder are more vulnerable to impairments in bonding due to the nature of their psychopathology, regardless of the occurrence of postpartum relapse. Careful follow-up including monitoring of mother-to-infant bonding of pregnant women with a history of bipolar spectrum disorder should be a standard to this vulnerable group of women. In addition, regardless of severity and mood episode relapse, an intervention to improve bonding could be beneficial for all mothers with bipolar spectrum disorder and their newborns.

## Introduction

Children of mothers with bipolar spectrum disorder are a high-risk group to develop psychopathology later in life. Offspring of parents with bipolar disorder are not only at an increased risk of developing a mood disorder later in life (1, 2), but also other disorders such as anxiety disorders (2) and sleeping disorders (3, 4). Bipolar disorder has an impact on general child developmental outcomes of the offspring (e.g., motor quality (5), cognition (visual and verbal memory, processing speed, attention) (6), intellectual functioning after the onset of bipolar disorder (7) and psychosocial development [e.g., work, interpersonal relations, recreation, global functioning (8)].

Offspring of bipolar mothers are at increased risk of psychopathology because of their genetic vulnerability, but environmental factors are thought to play an important role as well (3, 9–13). The often long and debilitating mood episodes of mothers with bipolar disorder might hamper their qualities as a caregiver and can have an impact on the environment of the child. This can start early in life with impaired mother-to-infant bonding (14). Mother-to-infant bonding is defined as “maternal feelings and emotions toward her infant” (15) (p1318). Impaired early mother-to-infant bonding has been found to be negatively associated with child behavioral and emotional development (16, 17), and higher levels of parenting stress during toddlerhood (18). In contrast to mother-to-infant bonding in women with bipolar disorder, mother-to-infant bonding in women with unipolar mood disorders has been well-studied. As expected, these studies have shown that depressive symptoms and depression, including postpartum depression, are associated with suboptimal early mother-to-infant bonding (19–24). Feeling inadequate, insecurity and negative cognitions are thought to influence bonding in mothers with depression (21).

Early mother-to-infant bonding in mothers with bipolar disorder has not been investigated, but a previous qualitative study described that mothers with bipolar disorder worried about potential problems in the relationship with their child. In this study, the majority of pregnant mothers with bipolar disorder were anxious to get a relapse postpartum potentially affecting them as a parent with some explicitly indicating concerns about bonding (25).

The aim of the current study was to examine mother-to-infant bonding 1 year postpartum in a prospective cohort of women with a bipolar spectrum disorder who were followed since pregnancy. We compared mother-to-infant bonding in mothers with a diagnosis in the bipolar spectrum [bipolar I (BD-I), bipolar II (BD-II), or bipolar Not Otherwise Specified (BD-NOS)] to mothers who participated in a large prospective population-based cohort, who were followed since pregnancy until 12 months postpartum in the Netherlands. As a next step, we aimed to investigate the association of mother-to-infant bonding with type of bipolar spectrum diagnosis and relapse within 12 months postpartum.

## Materials and Methods

### Participants and Procedure

#### Bipolar Spectrum Disorder Group

The participants of the current study were included in a longitudinal cohort study of women with BD. Women were recruited via specialized bipolar disorder treatment centers. Inclusion criteria were a primary diagnosis of bipolar spectrum disorder (bipolar I (BD-I), bipolar II (BD-II), or bipolar Not Otherwise Specified (BD-NOS). DSM IV and DSM V classification systems do not have a distinct disease category for women with a history of a single episode of mania or psychosis after delivery, but no episodes at other times and we therefore classified these women as bipolar NOS. All women were diagnosed by a psychiatrist prior to their current pregnancy. Other inclusion criteria were age ≥18, and a sufficient understanding of the Dutch language. Participants were approached by their psychiatrist and included between 2013 and 2018, and were prospectively followed from the third trimester of pregnancy until 1 year postpartum. Enrolled pregnant women were treated as usual. All participants were asked to complete several (online) questionnaires (including a bonding scale) throughout the perinatal period. Only women who completed follow-up assessment at 12 months postpartum were selected for inclusion in the current sample. Of the 95 women who received this follow-up questionnaire, 20 (21%) were lost to follow-up. This resulted in a total sample of 75 (79%) women to be included for analyses in the current study. There were no differences in characteristics between the 75 included women, and the women who completed baseline assessment. The participating 75 women with bipolar spectrum disorder included 34 (45.3%) women with a diagnosis of BD-I, 16 (21.3%) women with a diagnosis BD-II and, 25 (33.3%) women with BD not otherwise specified, including 24 women with a history of postpartum mania, but no episodes at other times. Of these women, 63 (85.1%) used psychotropic medication during their pregnancy. The study was approved by the Medical Ethics Committee at Erasmus University Medical Center, Rotterdam (MEC-2013-319). All women provided written informed consent.

#### Control Group

Additionally, a population-based control group of 1,419 Dutch-speaking perinatal women were examined [HAPPY study; Truijens et al. (26)]. These women filled in the same questionnaires administered at equivalent time points. Details regarding the design of this study have been described elsewhere (26). In short, exclusion criteria were a diagnosis of a severe psychiatric disorder (e.g., schizophrenia, borderline personality disorder and bipolar disorder), use of psychotropic medicine during pregnancy (e.g., antidepressants, SSRIs, benzodiazepines), twin or multiple pregnancies and a history of chronic disease (e.g., diabetes and thyroid dysfunction). This longitudinal cohort study followed women from the first trimester of pregnancy until 1 year postpartum. Throughout this period, women were asked to complete (online) questionnaires regarding their psychological and physical well-being. The study was approved by the Psychology Ethics Committee at Tilburg University (protocol number EC-2012.25), and reviewed by the Medical Ethics Committee at the Máxima Medical Center, Veldhoven. All women provided written informed consent.

### Measures

#### Mother-to-Infant Bonding

At 12 months postpartum, all mothers completed the Pre- and Postnatal Bonding Scale (19, 27), a self-report measure to assess feelings of bonding from a mother toward her infant. The scale consists of positive items assessing the core concept of love as a domain of bonding, and reflects positive feelings a mother might feel toward her child. Mothers were asked to rate items on a four-point Likert-type scale (0 = “not at all” to 3 = ‘very much’). Higher scores indicate more positive feelings of bonding. The PPBS showed adequate psychometric properties, with a Cronbach's alpha of 0.80 (19, 27).

#### Descriptive Characteristics

##### Recent Depressive Symptomology

At 12 months postpartum, mothers completed a screening question on their recent depressive symptomatology. Mothers indicated whether they had felt sad, miserable, or very anxious in the postpartum period for a period of at least 2 weeks, providing additional information on psychological well-being. Experience of recent depressive symptomatology was recoded into a dichotomous variable (yes/no).

##### Depressive Symptoms

In order to evaluate and compare the occurrence of depressive symptoms in both groups during late pregnancy (32 weeks) and early postpartum (6 weeks) we assessed depressive symptoms using the Dutch version of the Edinburgh Postnatal Depression Scale (EPDS) (28). The EPDS is a self-report measure and has been validated for use in non-postnatal women (29), as well as for use in a Dutch population, both during pregnancy (30) and postpartum (31). Items are rated on four-point Likert-type scales, with total scores ranging from 0 to 30. Higher scores indicate a greater level of depressive symptoms. At 12 months postpartum, the control group completed the EPDS, while women with a bipolar spectrum disorder completed this assessment when they indicated that they experienced recent depressive symptomatology. Furthermore, at 12 months postpartum, women were asked if they had experienced a relapse the 1st year postpartum and the severity of this episode was assessed using the EPDS (32).

##### Demographic Variables

Data regarding demographics and obstetric information were collected with online questionnaires in both mothers with a bipolar spectrum disorder and controls. In particular: age (in years), educational level (high: a Bachelor or Master's degree), having a partner (yes or no), and employment (yes or no). Furthermore, obstetric information was collected, such as parity (primiparous or multiparous), pregnancy intention (planned or unplanned), previous miscarriage or abortion (yes or no), and the gender of the child.

In the cohort of women with a bipolar spectrum disorder, information concerning the diagnosis and additional details with regard to a postpartum relapse (symptoms of depression, mania, psychosis) were obtained from their medical records.

### Statistical Analyses

Variables were compared between the control group and the bipolar spectrum disorder group using Chi-square tests for categorical variables and *t*-tests for continuous variables. If statistically significant, the strength of the relationship was expressed as Cramer's coefficients or Cohen's d and considered small or medium following conventional criteria.

First, we used multiple linear regression to assess the association between the maternal group (independent variable) and mother-to-infant bonding scores (dependent variable), adjusting for potential predetermined covariates based on previous studies (recent depressive symptomology, age, level of education, parity and unplanned pregnancy) (19).

Next, the group of women with a bipolar spectrum disorder was further analyzed to assess the association with mother-to-infant bonding within specific subgroups, such as primary diagnosis (bipolar disorder I (BD-I), bipolar disorder II (BD-II), or history of postpartum mania and perinatal relapse (yes/no). Relapse rate was assessed and compared between subgroups of primary diagnosis using Chi-square tests. Depressive symptoms during pregnancy and early postpartum were also compared between women with and without a relapse, using *t*-test. Multiple linear regression analyses were also conducted to assess these associations. For multiple linear regression analyses, the BD-I, BD-II, and postpartum mania group were coded into dichotomous variables, while the variable age was centered on the mean. Statistical analyses were performed using IBM SPSS version 26.0.

## Results

The demographic and obstetric characteristics of the bipolar spectrum disorder group and the control group are shown in Table 1. Differences were found with regard to age and parity between the two groups, with women with bipolar spectrum disorder being slightly older (medium effect size) and more often multiparous. The control group had a paid job more often. Compared to the control group, more women with bipolar spectrum disorder reported that their pregnancy was unplanned. Apart from age, all differences had a small effect size. The groups did not differ with regard to level of education, previous miscarriage or abortion, having a partner, and prenatal and postnatal depressive symptoms.

**Table 1:** Demographic, psychiatric and obstetric characteristics of the participating women.

	**Bipolar spectrum disorder (*****n*** **= 75)**				**Control group (*****n*** **= 1,419)**				
	***N***	**%**	**Mean (SD)**	**Range**	***N***	**%**	**Mean (SD)**	**Range**	***p***
Demographics									
Age			32.8 (4.1)	25–43			30.4 (3.6)	19–43	<0.001
High level of education	52	69.3			973	67.6			0.714
Paid job	43	58.1			1,307	94.2			<0.001
Partner	73	98.6			1,379	99.2			0.603
Disease characteristics									
Bipolar Disorder I	34	45.3			–	–	–	–	–
Bipolar Disorder II	16	21.3			–	–	–	–	–
Bipolar Disorder NOS	25	33.3			–	–	–	–	–
Psychotropic medication	63	85.1			–	–	–	–	–
Obstetric characteristics									
Parity									<0.001
Primiparous	21	28.8			740	52.9			
Multiparous	52	71.2			660	47.1			
Planned pregnancy	62	84.9			1,328	95.6			<0.001
Previous miscarriage/abortion	19	25.7			341	24.6			0.829
Gender Child									
Girl	38	50.7			713	50.7			0.965
Boy	37	49.3			687	49.3			
EPDS score									
32 weeks pregnancy			4.8 (5.2)	0–22			4.8 (4.0)	0–23	0.967
6 weeks postpartum			5.3 (5.3)	0–21			4.8 (4.4)	0–25	0.394
Bonding score									
12 months postpartum			12.5 (2.1)	8–15			13.8 (1.7)	5–15	<0.001
Recent depressive symptomatology	29	38.7			35	2.5			<0.001

*SD, standard deviation; level of education, low: primary education or secondary pre-vocational education, medium: secondary education or vocational education, high: Bachelor or Master's degree; EPDS, Edinburgh Postnatal Depression Scale; higher bonding scores indicate more positive feelings of bonding*.

Furthermore, of the mothers with a bipolar spectrum disorder, 29 (38.7%) experienced recent depressive symptomatology, while only 35 (2.5%) of the control group experienced these symptoms [$X_{\left(1,\;1,494\right)}^{2}$ = 227.67, *p* < 0.00, Phi = −0.390, medium effect size].

### Group Differences in Mother-to-Infant Bonding

#### BD Group vs. Control Group

Mean bonding scores differed between groups (*t* = 5.17, *p* < 0.001, Cohen's d = 0.67, medium effect size), with women in the bipolar spectrum disorder group reporting lower mother-to-infant bonding scores (M = 12.46, SD = 2.13) compared to the control group (M = 13.76, SD = 1.68). Results of the multiple linear regression model predicting mother-to-infant bonding showed that the total model was significant, F_(6, 1, 445)_ = 15.86, *p* < 0.001, *R*^2^ = 0.062. Bipolar spectrum disorder was independently associated with poorer mother-to-infant bonding scores compared to the control group after adjustment for recent depressive symptomatology, age, education, parity and unplanned pregnancy (β = −0.11, SE = 0.23, *p* < 0.001), see Table 2.

**Table 2:** Multiple regression predicting mother-to-infant bonding (*N* = 1,445).

	**B (SE)**	**β**	***t***	***p***
Bipolar spectrum disorder	−0.89 (0.23)	−0.11	−3.95	<0.001
Recent depressive symptomatology	−0.76 (0.24)	−0.09	−3.18	0.002
Maternal age	−0.002 (0.01)	−0.01	−0.17	0.869
High level of education	−0.49 (0.10)	−0.13	−4.85	<0.001
Multiparous	−0.33 (0.09)	−0.10	−3.57	<0.001
Unplanned pregnancy	0.03 (0.21)	0.004	0.14	0.888

*B, unstandardized regression coefficient; SE, standard error; β, standardized regression coefficient*.

#### Group Differences Among Mothers With Different Types of Bipolar Spectrum Disorder

Mothers with a BD-I or BD-II diagnosis reported higher mean bonding scores (more positive bonding) 12 months postpartum (M = 12.76, SD = 2.09, and M = 12. 88, SD = 1.96, respectively) compared to women with a history of postpartum mania (M =11.67, SD = 2.14). (*t* = 1.95, *p* = 0.056, Cohen's d = 0.52, medium effect size and, *t* = 1.81, *p* = 0.079, Cohen's d = 0.59, medium effect size, respectively). There was no difference in mean bonding scores between the BD-I and BD-II group (*t* = 0.18, *p* = 0.86). After adjustment for recent depressive symptomatology, age, education, parity and unplanned pregnancy, the primary diagnosis was not independently associated with poorer bonding scores (BD-I: β = 0.21, SE = 0.68, *p* = 0.191 and BD-II: β = 0.17, SE = 0.85, *p* = 0.306).

### Effect of Postpartum Relapse on Mother-to-Infant Bonding

In total, 27 (37.5%) women had a relapse within 12 months after giving birth, defined as a depressive episode, hypo(-mania) and/or psychosis. There was a significant difference in mean EPDS scores at 6 weeks postpartum between women with a relapse (M = 7.72, SD = 5.83) and women without a relapse (M = 4.10, SD = 4.58) (*t* = 2.79, *p* = 0.007, Cohen's d = 0.72, medium effect size). With regard to the primary diagnosis, 10 (31.3%) of the women with BD-I, 9 (56.3%) of the women with BD-II and, 8 (34.8%) of the women with a history of postpartum mania relapsed during the current postpartum period.

Mean bonding scores did not differ between women with (M = 12.22, SD = 2.21) and without a relapse (M = 12.64, SD = 2.08) (*t* = 0.82, *p* = 0.418).

## Discussion

The current study aimed to assess mother-to-infant bonding in mothers with a bipolar spectrum disorder. The present study showed that women with bipolar spectrum disorder showed worse bonding scores at 12 months postpartum compared to postpartum population-based control women, after controlling for several potential confounders. The type of the primary diagnosis (bipolar I disorder, bipolar II disorder or bipolar NOS, including women with a history of postpartum mania) was not associated with impaired bonding. The occurrence of relapse in the perinatal period did not influence the bonding score, which was unexpected.

Our findings add to the knowledge that women with bipolar spectrum disorder and their offspring are a vulnerable and high-risk group, providing a first indication of a suboptimal mother-to-infant bonding early in life. Possibly, women with bipolar spectrum disorder have increased fears of failure with regard to bonding, as well as feelings of insecurity, which may impact their perceptions of the bond with their child. These worries have been highlighted in the results of a recent qualitative study, showing that mothers with a bipolar disorder feared their mood episodes would affect bonding with their child (e.g., not wanting their child) (25).

The postpartum relapse rate in our study was high (37.5%) but in line with our prior meta-analysis in which we reported an overall postpartum relapse risk of 35% (95% CI = 29, 41) (33). Interestingly, relapse during the current postpartum period did not affect bonding scores in women with a bipolar spectrum disorder. Our findings could imply that women with a bipolar spectrum disorder are more vulnerable to impairments in bonding due to the nature of their disorder, regardless of relapse. Similar to mothers with depression, feelings of inadequacy, insecurity and negative cognitions (21) could influence bonding in mothers with a bipolar spectrum disorder. Due to the small sample of women with relapse, we were unable to assess differences in bonding regarding the severity, timing, and symptomatology of relapse (depressive symptoms, mixed episode or psychosis/mania). Studies that have compared mother-to-infant bonding in patients with postpartum depression and postpartum psychosis who had been admitted to a mother-baby unit, found that especially women with postpartum depression perceive impairments in bonding (21, 22). Notably, these studies did not include a population of patients diagnosed with a bipolar spectrum disorder, nor a control group, making it difficult to compare them with the findings of the current study.

The current study has several strengths and limitations. The strength of the study is the case-control design of participants who were longitudinally studied during pregnancy and in the postpartum period. Our study also has limitations that should be considered when interpreting the results. First, the bonding questionnaire is a short screening instrument examining the core concept of mother-to-infant bonding (core concept of love), and shows good psychometric properties and reliability (19, 27). Future research is needed to more extensively assess differences in various components of early bonding (e.g., rejection, anxiety about care) in mothers with a bipolar spectrum disorder. A major limitation of the study is the lack of assessment on current mood status. Depressive symptoms were assessed using the EPDS at 12 months postpartum, in those women with a relapse only. Although current symptomatology was not measured using a standard assessment (e.g., EPDS), recent depressive symptomatology during the postpartum period was assessed using a screening question, which was included in the analyses as a covariate. Mothers with a bipolar spectrum disorder reported poorer mother-to-infant bonding scores, even after controlling for depressive symptomatology. Furthermore, the bonding scores did not differ between mothers with a bipolar spectrum disorder who experienced recent depressive symptomatology compared to mothers without these symptoms. For the screening question, women reflected on their depressive symptomatology over the entire postpartum period. Even so, we expect that a telescoping effect may have occurred (34), causing women to have reflected on more recent symptoms when completing this screening question, rather than symptoms occurring more in the past (e.g., early postpartum). This may have led to participants reporting more current symptomatology. Nonetheless, we recommend that this effect is further investigated and validated in other studies and groups. Another limitation of the current study is that both women in the control cohort and the bipolar spectrum disorder cohort were more often highly educated compared to the general Dutch population (35) and were predominantly Caucasian which hampers generalizability of our results. Moreover, 21% of women with a bipolar spectrum disorder were lost to follow-up, and therefore did not complete bonding assessment at 12 months postpartum, leading to incomplete follow-up of these women in the study over time. Lastly, the diagnosis of an episode of relapse was made by the treating psychiatrist and not by a formal diagnostic interview. The study was designed to limit the burden of study participation in a vulnerable group of pregnant and postpartum women with the aim to recruit a representative sample of bipolar women in terms of disease severity, and not only women with relatively mild or stable bipolar disorder who are willing to do extra in person diagnostic interviews for research purposes.

The current findings suggest that women with a history of bipolar spectrum disorder prior to the current pregnancy, regardless of relapse during the current postpartum period, are at increased risk for impaired mother-to-infant bonding. Our findings suggest that incorporating aspects of bonding in interventions for mothers with a history of a bipolar spectrum disorder could be valuable, irrespective of a relapse. For example, Practical Resources for Effective Postpartum Parenting is an intervention for women at risk for postpartum depression that focuses on the mother-infant dyad, and had positive outcomes for the mother, child and the mother-child relationship (36). Next, findings of a meta-analysis showed that maternal-child interaction guidance could be a promising intervention for mothers with postpartum depression, with regard to parenting and child outcomes (37). Future studies should address the effectiveness of such interventions for bipolar disorder mother-infant dyads. Suboptimal bonding is a risk factor for negative behavioral and emotional outcomes in children (16, 17). It is therefore important to integrate interventions to improve bonding in the treatment of mothers with a bipolar spectrum disorder. It is also of importance that polyclinic and obstetric health care facilities provide follow-up for parents with mental illness and their newborn. Especially because offspring of parents with bipolar disorder are at ultra-high risk for developing a mood disorder and other disorders later in life (1–3). Mother-to-infant bonding is a process that can already begin prenatally (38) and future research could focus on the best timing and type of intervention to improve bonding.

## Data Availability Statement

The data can be requested from the authors. Requests to access the datasets should be directed to m.g.b.m.boekhorst@uvt.nl.

## Ethics Statement

The studies involving human participants were reviewed and approved by Medical Ethics Committee at Erasmus University Medical Center, Rotterdam MEC-2013-319 Psychology and Ethics Committee at Tilburg University EC-2012.25. The patients/participants provided their written informed consent to participate in this study.

## Author Contributions

MB contributed to the hypotheses, data analyses and wrote the paper. AB contributed to the hypotheses and collaborated in writing and editing of the paper. MH and VP contributed to the hypotheses and collaborated in writing and editing of the paper. VB is the principal investigator and contributed to funding acquisition, material preparation, process of data collection, hypotheses, data analyses and collaborated in writing and editing of the paper. All authors read and approved the final version of the manuscript.

## Conflict of Interest

The authors declare that the research was conducted in the absence of any commercial or financial relationships that could be construed as a potential conflict of interest.
